# Automatic Programmable Bioreactor with pH Monitoring System for Tissue Engineering Application

**DOI:** 10.3390/bioengineering9050187

**Published:** 2022-04-25

**Authors:** Suruk Udomsom, Apiwat Budwong, Chanyanut Wongsa, Pakorn Sangngam, Phornsawat Baipaywad, Chawan Manaspon, Sansanee Auephanwiriyakul, Nipon Theera-Umpon, Pathinan Paengnakorn

**Affiliations:** 1 Biomedical Engineering Institute, Chiang Mai University, Chiang Mai 50200, Thailand; suruk_u@cmu.ac.th (S.U.); apiwat_bouthwong@cmu.ac.th (A.B.); chanyanut_w@cmu.ac.th (C.W.); pakorn.sangngam@cmu.ac.th (P.S.); phornsawat.b@cmu.ac.th (P.B.); chawan.m@cmu.ac.th (C.M.); sansanee.a@cmu.ac.th (S.A.); nipon.t@cmu.ac.th (N.T.-U.); 2 Department of Computational Engineering, Faculty of Engineering, Chiang Mai University, Chiang Mai 50200, Thailand; 3 Department of Electrical Engineering, Faculty of Engineering, Chiang Mai University, Chiang Mai 50200, Thailand; 4 Center of Excellence for Innovation in Analytical Science and Technology, Chiang Mai University, Chiang Mai 50200, Thailand

**Keywords:** bioreactor, tissue engineering, automation, sensor

## Abstract

Tissue engineering technology has been advanced and applied to various applications in the past few years. The presence of a bioreactor is one key factor to the successful development of advanced tissue engineering products. In this work, we developed a programmable bioreactor with a controlling program that allowed each component to be automatically operated. Moreover, we developed a new pH sensor for non-contact and real-time pH monitoring. We demonstrated that the prototype bioreactor could facilitate automatic cell culture of L929 cells. It showed that the cell viability was greater than 80% and cell proliferation was enhanced compared to that of the control obtained by a conventional cell culture procedure. This result suggests the possibility of a system that could be potentially useful for medical and industrial applications, including cultured meat, drug testing, etc.

## 1. Introduction

Tissue engineering has been developed with the aim of increasing the opportunity of substituted tissue production for restoring or regenerating tissue and organ function. In recent years, development in tissue engineering technology has grown rapidly, and it has been widely applied to various fields apart from the medical area, such as organs-on-chip, bioelectronic devices, cultured meat, etc. [1,2]. The production of tissue engineering mainly relies on the cell culture process. Therefore, quality control of cell culture conditions is essential for tissue engineering research [3]. The critical factors for mammalian cell culture include temperature, pH, O_2_ and CO_2_ concentrations, humidity, nutrition and growth factor content, etc. [4,5,6]. In a laboratory, the cell culture process is time-consuming and laborious. Therefore, in order to increase cell production capacity and reduce human error, an automatic and programmable bioreactor for tissue engineering equipped with cell condition monitoring could reduce time and workload for researchers. Also, the monitoring system could provide useful data for profiling cell growth and enhancing the productivity of the process [7,8].

Nowadays, automation of analytical systems has been utilized in many areas, such as in clinical, pharmaceutical, and biomedical applications [9,10,11]. The automatic system has played an important role in both qualitative and quantitative analysis. Various analytical techniques have been employed (for example, electrochemical, optical, and mass spectroscopic methods). The robotic automation could offer high precision and high system throughput while reducing analysis costs. A study of integrated measurement tools or sensors for cell metabolism monitoring through cell culture procedures has been widely of interest for a decade. There are several types of sensors integrated into general cell cultivation, bioreactors, and labs on a chip, such as oxygen sensors, pH sensors, and glucose-lactate biosensors [12]. The role of a cell culture monitoring tool indicates the viability of the cells, the consumption of nutrients, the nutrient yield in fermentation media, and the effect of drugs [13,14,15].

There have been several lab-on-chip developments for cell culture and other biomedical applications. In organ-on-chip, the microfluidic platforms allowed co-culture of different cells to mimic the organ systems for drug testing [16]. Many studies involved real-time monitoring of cell activity and gene expression [17,18,19]. They provided a useful platform for cellular investigation. However, the number of cells on the lab-on-chip is limited and the results from microscopic experiments could be different, so scale-up could be challenging [20]. Moreover, there are limitations concerning the complications and availability of fabrication technology for general research laboratories.

In this work, an automatic programmable bioreactor with a pH monitoring system was developed. All system components were made in-house with computer-aided design (CAD) and 3D-printing techniques. The bioreactor consists of cell culture chamber, syringe pump, selector, pH sensor, and a controlling program. The developed system was customizable for different cell types, and the program was customizable for different culture conditions.

Two types of 3D printing techniques were used. The main 3D printing technology used is liquid crystal display (LCD)-based stereolithography (SLA) that is based on photopolymerization of monomer and oligomer resin layer by layer [21]. This 3D printing technique has the advantage of high-resolution printing, but the size of printing object is limited by the LCD size, so it was chosen for making small and detailed components such as selector and pH sensor case. On the other hand, fused deposition modeling (FDM) 3D printing was employed for large and less-detailed components such as a syringe pump.

The obtained data from the pH monitoring system could also be tracked for inspection. The developed system was demonstrated with cell culture of the L929 cell line in comparison with the conventional cell culture procedure.

## 2. Materials and Methods

### 2.1. Design and Construction of Bioreactor

**Figure 1:**
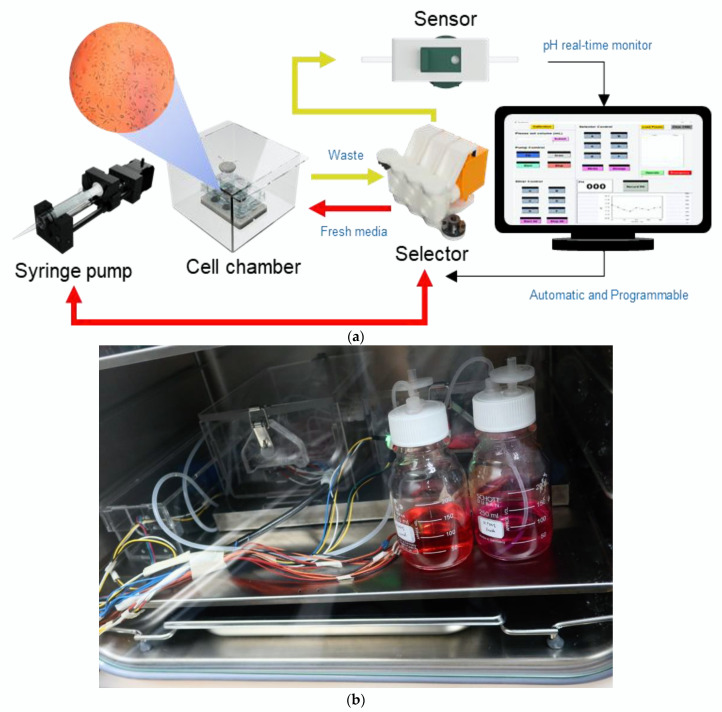
A bioreactor with a pH monitoring system: (**a**) a schematic diagram showing components of the bioreactor; (**b**) a photo of the bioreactor inside an incubator.

The bioreactor consists of cell culture chamber, syringe pump, selector, pH sensor and controlling program as shown in Figure 1. The hardware parts were designed using a CAD program (Fusion 360, Autodesk Incorporation, San Rafael, CA, USA). The details of each component were described here.

#### 2.1.1. Cell Culture Chamber

**Figure 2:**
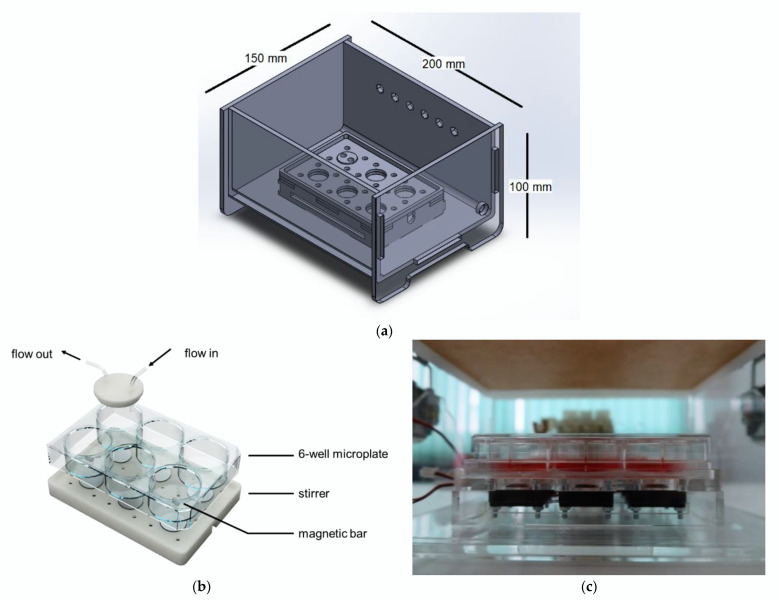
Cell culture chamber: (**a**) a CAD file of a cell culture chamber design with a well plate holder; (**b**) a CAD file showing 6-well plate with PDMS lids and 6 micro-stirrers; (**c**) a side view of a cell culture on a 6-well plate inside a cell culture chamber.

A cell culture chamber was designed to accommodate one standard cell culture well plate size of 128 mm × 86 mm (length × width). For a prototype, a 6-well plate was used as a model, as shown in Figure 2. The main body and lid of the chamber were made of transparent acrylic with a thickness of 5 mm that was sterilized with ethanol and UV radiation before use. Each well was covered with a piece of polydimethylsiloxane (PDMS; Dow Inc., Midland, MI, USA) and a flow channel was connected to a selector outside of the chamber via a luer connector. The cell culture chamber is also equipped with 6 micro-stirrers that can be used for suspension cell culture.

#### 2.1.2. Syringe Pump

**Figure 3:**
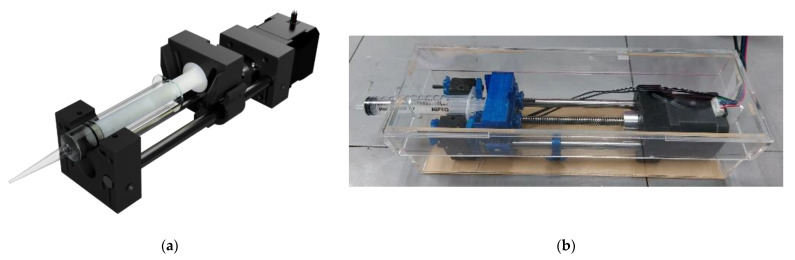
Syringe pump: (**a**) a CAD file of syringe pump design; (**b**) a photo of syringe pump with a 10-mL syringe in an acrylic box.

A syringe pump consists of a stepper motor and a syringe holder. A stepper motor (NEMA17, Shenzhen, China) was employed, as shown in Figure 3. A syringe holder was constructed using fused deposition modeling (FDM) 3D printing (in-house) with acrylonitrile butadiene styrene (ABS) filament (eeSun, Shenzhen, China). The syringe pump was designed to house a disposable sterile 10-mL syringe (Nipro (Thailand) Co Ltd., Ayutthaya, Thailand) that connected with a female luer adaptor and a silicone tubing (inner diameter of 2 mm, Runze Fluid, Nanjing, China). The syringe pump was placed in a clear acrylic box and sterilized with ethanol and UV radiation before use.

#### 2.1.3. Selector

**Figure 4:**
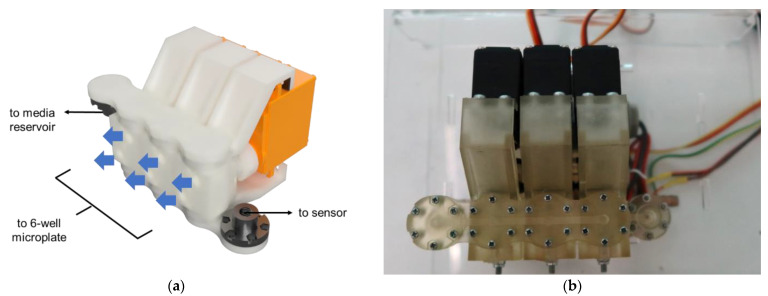
Selector: (**a**) a CAD file of a selector design; (**b**) a photo of a selector with 3D-printed channels.

A selector acts as a media flow switch between a syringe pump, a cell culture well, a media reservoir, and sensors. It has 3 servo motors (MG996R, Shenzhen, China) that rotate to open and close the desired channel as illustrated in Figure 4. The rotating parts and flow channels were constructed using LCD-based 3D printing (ANYCUBIC Photon Mono X, Hong Kong Anycubic Technology, Hong Kong, China) with acrylic-based resin (eResin PLA biophotopolymer resin, eSun, Shenzhen, China). The outlet was fitted with PTFE flangeless fittings and TPFE tubing (inner diameter of 1 mm, Runze fluid, Nanjing, China). The selector was placed in a clear acrylic box and sterilized with ethanol and UV radiation before use.

#### 2.1.4. pH Monitoring System

**Figure 5:**
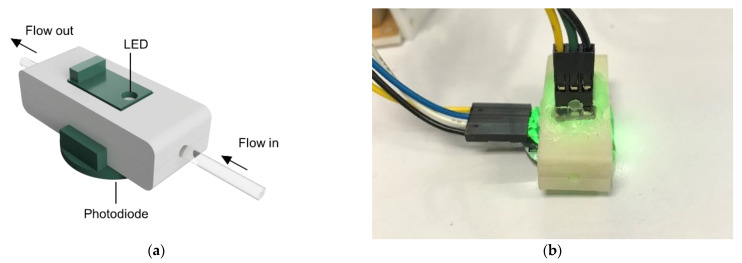
On-line pH monitoring system. (**a**) a CAD file of a pH sensor; (**b**) a photo of a pH sensor with green LED.

An on-line pH monitoring system was based on colorimetric measurement of cell culture media containing phenol red indicator. As shown in Figure 5, a pH monitoring system consists of an LED light source, an ambient light sensor (TEMT 6000, Vishay Intertechnology, Malvern, PA, USA), and a tubing holder that allows for non-contact measurement. The tubing holder is designed to fit a PTFE tube and was constructed using LCD-based 3D printing (ANYCUBIC Photon Mono X, Hong Kong Anycubic Technology, China) with opaque acrylic-based resin (eResin PLA biophotopolymer resin, eSun, Shenzhen, China).

#### 2.1.5. Controlling Program

**Figure 6:**
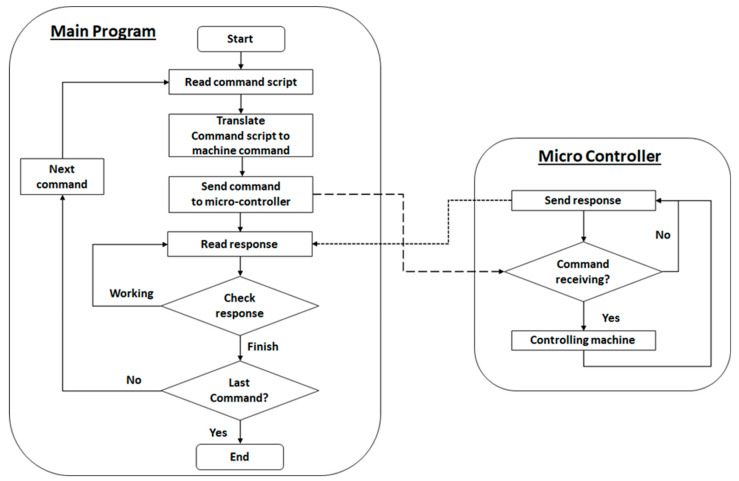
Schematic diagram showing workflow of controlling program.

**Figure 7:**
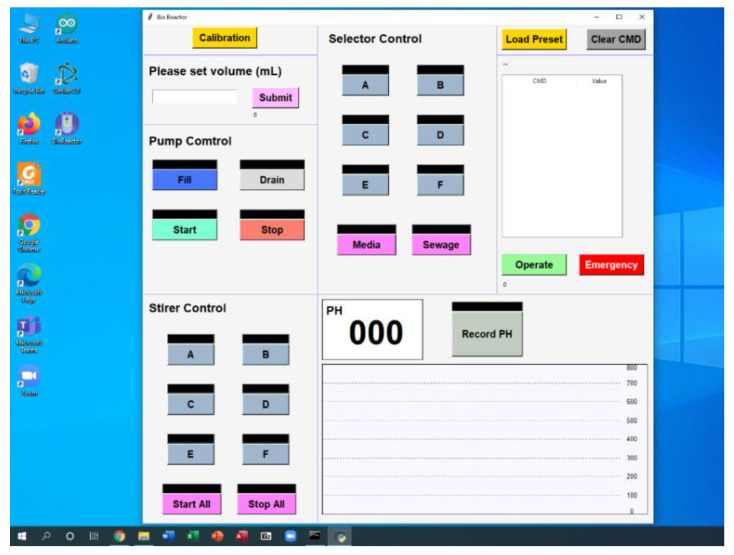
A screenshot of user interface of controlling program.

The bioreactor is controlled by simple software as shown in Figure 6 and Table 1. We developed software for controlling syringe pump, which can set the volume of culture media fed into and out of the culture chamber. For the selector, the software can control the selector to select the channel of each culture well, fresh media, and waste. Also, this software can control the stirrer of each well plate for mixing solution in each well. The pH monitoring system readings are recorded and a graph was plotted on the graphic user interface (GUI). In addition, we can write the preset script of the culture condition in advance and this software can control the bioreactor automatically. The GUI of this software is shown in Figure 7.

### 2.2. Bioreactor Setup

Before using the developed bioreactor, a syringe pump was calibrated by weighting water flew out at different flow rates using a four-digit balance. A pH monitoring system was calibrated using culture media with different pH values, which were predetermined with a laboratory pH meter.

A set of PDMS lids, containers for fresh media and waste, and silicone tubing were sterilized using an autoclave beforehand.

All components except a microcontroller and a notebook with a controlling program were sterilized with ethanol and then UV radiation for 15 min in a biosafety cabinet before use. A 6-well plate containing cell culture was placed in a chamber and a media reservoir and waste container were connected to a selector in a biosafety cabinet. A bioreactor with cell culture was then placed in an incubator.

### 2.3. Bioreactor Performance Test

The performance of the developed bioreactor was tested by measuring the cell viability and cell proliferation rate of cells cultured in the bioreactor compared with that of a conventional cell culture procedure. A mouse fibroblast cell line (L929: NCTC clone 929, JCRB cell bank, Osaka, Japan) was used as a model for this test. The cells were cultured in Dulbecco’s modified Eagle medium (DMEM, Thermo Fisher Scientific, Waltham, MA, USA) with 10% fetal bovine serum (FBS, Thermo Fisher Scientific, USA) and 1% antibiotic-antimycotic (100X, Thermo Fisher Scientific, USA).

#### 2.3.1. Cell Viability Testing

The L929 cells were seeded in a 6-well plate at 1 × 10^5^ cells per well. The well plate was then placed in the bioreactor and was incubated at 37 °C with 5% CO_2_ and a humidified atmosphere for 48 h. The control group was place in the same incubator. The cell viability was measured using an MTT assay [22].

#### 2.3.2. Cell Proliferation Assay

The L929 cells were seeded in a 6-well plate at 7 × 10^4^ cells per well with 5 mL of complete media and then cultured automatically in the bioreactor for 72 h using a pre-set program where 2 mL of cultured media was disposed of and refilled every 6 h. At 24 h and 72 h, the cells were trypsinized using 0.25% trypsin-EDTA solution (Thermo Fisher Scientific, USA) and the number of cells was counted with an automated cell counter (Countess II, Thermo Fisher Scientific, USA). The obtained proliferation rate was compared to that obtained from conventional cell culture with the same starting cell numbers.

### 2.4. pH Monitoring System Test

The pH monitoring system was tested during the incubation of L929 cells for 48 h. The program was set to refresh 2 mL of cell culture media every 6 h.

## 3. Results

### 3.1. Syringe Pump Calibration

**Figure 8:**
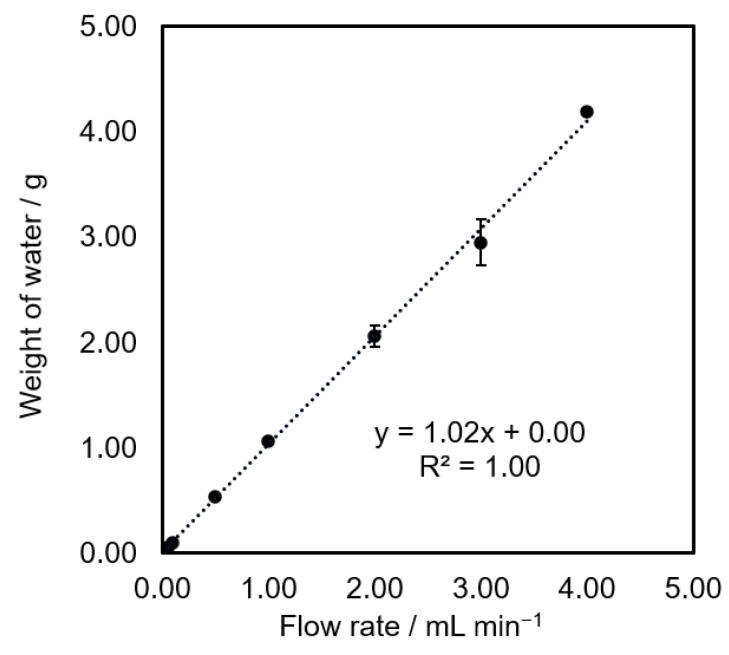
A calibration graph of an in-house syringe pump, *n* = 3.

A calibration of the syringe pump with a 10 mL syringe was performed. The different flow rates were set for one minute and the flow out water was collected and precisely weighted. In Figure 8, the calibration plot showed that the mass of flow out water is correlated with the set flow rate. This result indicates that the flow rate of the calibrated syringe pump was accurate.

### 3.2. pH Calibration

**Figure 9:**
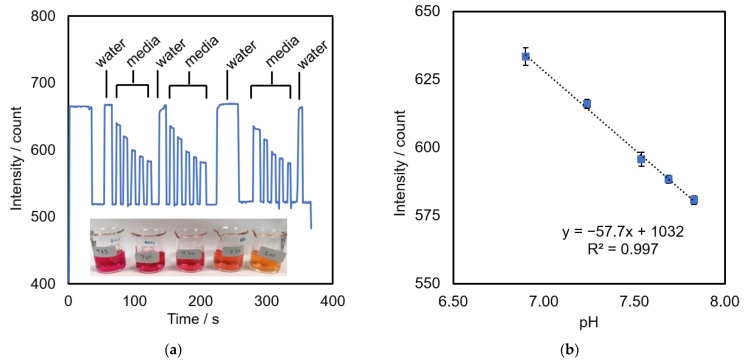
Optical pH sensor: (**a**) effect of pH on green intensity measured by flowing DMEM media at various pH (shown in inset) thought a sensor; (**b**) a calibration plot between pH of DMEM media and green intensity, *n* = 3.

The pH monitoring system was calibrated with DMEM media, which is used for the culture of the L929 cell model. The media contains a phenol red indicator that changes color in the range of 6.8–8.2 from yellow to pink, respectively, as shown in Figure 9a. An increase in the pH of the media caused an increase in the red color intensity, which absorbed more green light and will be detected by the sensor. Figure 9b shows a calibration plot obtained from DMEM at a pH range between 6.90 and 7.83; green intensity = −57.7 (pH) + 1032, *n* = 3.

### 3.3. Cell Viability—MTT Assay

**Figure 10:**
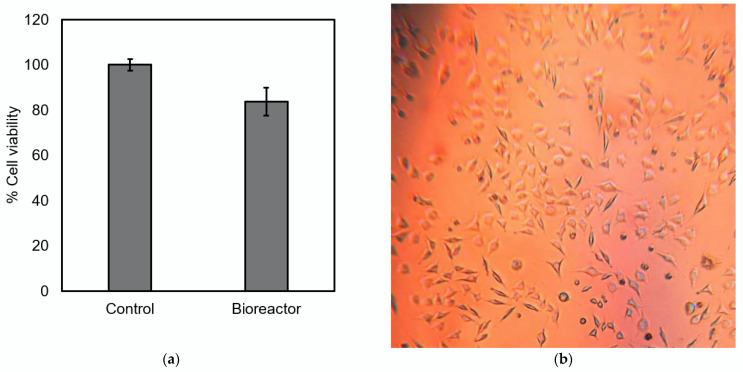
Cell viability of L929 incubated in the developed bioreactor: (**a**) percent cell viability obtained from MTT assay with respected to control group (*n* = 6); (**b**) a microscopic image of cells after incubation in the bioreactor for 48 h.

The cell viability of L929 cells was measured after incubation in the developed bioreactor for 48 h. Figure 10a shows that the cell viability of the L929 cell in the bioreactor was greater than 80% when compared to the control obtained through conventional cell culture. In addition, no morphologic change was observed, as shown in Figure 10b. These results suggest that the developed bioreactor including setup conditions and sterilization process did not cause a cytotoxic effect on the cells.

### 3.4. Cell Proliferation Assay

**Figure 11:**
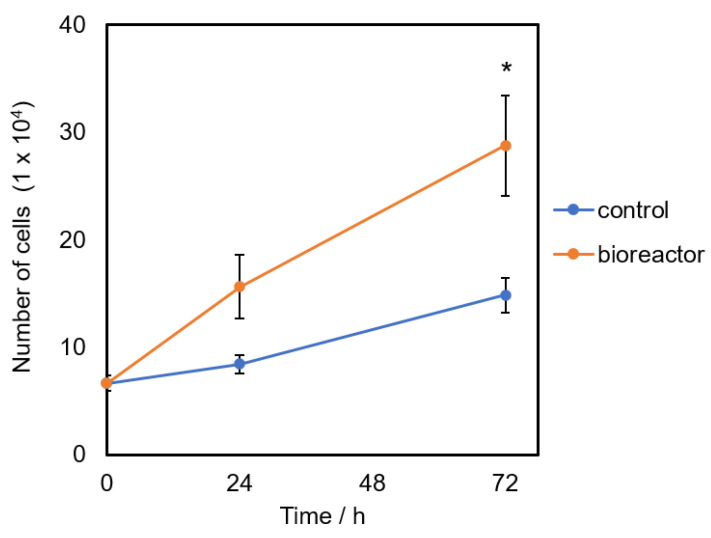
Cell proliferation of L929 incubated in the developed bioreactor. The number of cells was counted at 24 and 72 h after incubation and the data were analyzed by two-way ANOVA and post-hoc using R program. * Statistical significance was set at *p* < 0.05, *n* = 3.

An automatic cell culture in the developed bioreactor was demonstrated. Figure 11 shows cell proliferation from L929 cells cultured in the bioreactor with the program to refresh the media every 6 h automatically compared to that cultured parallelly in a conventional manner (no media changed). It was found that the number of cells in the bioreactor increased significantly at 72 h in comparison with the number of cells in the control plate. The results suggested that the automation of regular partial media change could help enhance the cell growth in the bioreactor.

### 3.5. pH Monitoring Results

The pH measurement of the cultured media was displayed in real-time and recorded as .CSV file for further analysis. The pH was measured by the pH sensor located on the channel before the waste container, indicating the pH of the used media. In general, acidic conditions could inhibit cell growth. A change in pH could reflect the cell condition, such as a lower pH suggesting cell death or bacterial contamination.

**Figure 12:**
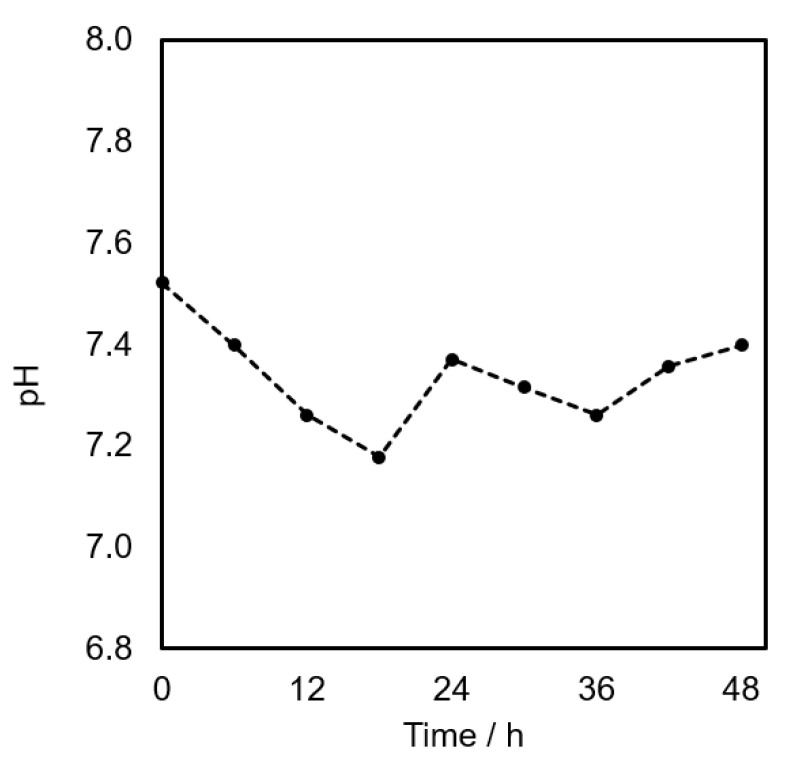
pH changes during L929 cell incubation in the developed bioreactor.

Figure 12 shows a plot of the pH change with time during a 48-h cell culture of L929 cells in the developed bioreactor. It was found that a program set for regular media change was able to maintain the pH of the cell culture within 7.4 ± 0.2, which is suitable for fibroblast cell line [23]. This could be related to the higher cell proliferation rate in the bioreactor observed in Figure 11.

## 4. Discussion

In this work, we developed an automatic programmable bioreactor with a pH monitoring system that was suitable for tissue engineering cell culture. This bioreactor’s components can be controlled via programable script to the desired cell culture condition, and this system can operate automatically. This system helps to reduce time and labor compared to manual cell culture.

For pH sensor, we applied a colorimetry technique to monitor the pH of the cell culture on-line. This sensor was compact and easy to use. In addition, non-contact measurement has an advantage in real-time monitoring, and there is no contamination to cell culture. The data were collected and can be analyzed for cell culture condition scripts next time. In future work, we plan to use machine learning methods, for example, the deep learning method [24,25], to optimize and find the suitable parameters in controlling bioreactors in order to enhance the productivity of the cell culture in the bioreactor.

Furthermore, this prototype can be applied to culture cells for a long period to study cell differentiation and tissue formation, which could be potentially useful for medical and industrial applications, including cultured meat, drug testing, etc. For example, this system could be used to study muscle cell differentiation and determine optimal conditions for cultured meat production. For drug testing, the bioreactor could be programmed to vary the dose or frequency of the treatment. In the future, an additional analytical system could be added to the bioreactor to detect disease-specific biomarkers or to monitor other biomolecules such as glucose and lactate dehydrogenase, which would indicate cells’ activity.

## Figures and Tables

**Table 1:** List of commands for controlling bioreactor.

Command	Value	Description
Loop	number	Set number of scripts to repeat
Vol	number	Set volume of syringe pump to fill or drain (mL)
Goto	number	Jump to a line number that set of the script
Wait	hh:mm:ss	Set delay time
Dir	F or B	Set syringe pump direction (Forward or Backward)
Ch	Selector channel (A or B or C or else)	Select selector channel
Stir	Stirrer channel (A or B or C or else)	ON/OFF selected stirrer
Start		Starting syringe pump
Msgbox	Text	Displays the specified text in the message box

## Data Availability

The data presented in this study are available on request from the corresponding author.
